# Provisioning the Ritual Neolithic Site of Kfar HaHoresh, Israel at the Dawn of Animal Management

**DOI:** 10.1371/journal.pone.0166573

**Published:** 2016-11-30

**Authors:** Jacqueline S. Meier, A. Nigel Goring-Morris, Natalie D. Munro

**Affiliations:** 1 Department of Anthropology, University of Connecticut, Storrs, Connecticut, United States of America; 2 Institute of Archaeology, Hebrew University, Jerusalem, Israel; Chinese Academy of Sciences, CHINA

## Abstract

It is widely agreed that a pivotal shift from wild animal hunting to herd animal management, at least of goats, began in the southern Levant by the Middle Pre-Pottery Neolithic B period (10,000–9,500 cal. BP) when evidence of ritual activities flourished in the region. As our knowledge of this critical change grows, sites that represent different functions and multiple time periods are needed to refine the timing, pace and character of changing human-animal relationships within the geographically variable southern Levant. In particular, we investigate how a ritual site was provisioned with animals at the time when herd management first began in the region. We utilize fauna from the 2010–2012 excavations at the mortuary site of Kfar HaHoresh—the longest continuous Pre-Pottery Neolithic B faunal sequence in the south Levantine Mediterranean Hills (Early–Late periods, 10,600–8,700 cal. BP). We investigate the trade-off between wild and domestic progenitor taxa and classic demographic indicators of management to detect changes in hunted animal selection and control over herd animal movement and reproduction. We find that ungulate selection at Kfar HaHoresh differs from neighboring sites, although changes in dietary breadth, herd demographics and body-size data fit the regional pattern of emerging management. Notably, wild ungulates including aurochs and gazelle are preferentially selected to provision Kfar HaHoresh in the PPNB, despite evidence that goat management was underway in the Mediterranean Hills. The preference for wild animals at this important site likely reflects their symbolic significance in ritual and mortuary practice.

## Introduction

The growing body of zooarchaeological and genetic evidence from Southwest Asia increasingly supports multi-regional trajectories toward domestication [1–4]. In particular, the process of animal domestication is marked by significant variation among regional zones, with regards to the timing of its first appearance and pace of change, and the type of faunal evidence available to document its early stages [5,6]. Among the many explanations for this variation are differing rates of the spread of agricultural knowledge or animals, localized adaptations of management strategies, proximity to migration and trade routes, and the type of vegetation available for graze [7,8]. Most researchers agree that some form of animal management, at least of goats, had emerged in the southern Levant by the Middle Pre-Pottery Neolithic B (MPPNB) period, although whether it occurred locally or diffused from the north, is less widely agreed upon (see below). More faunal studies are needed from sites spanning PPNB periods to illuminate how local processes of animal management unfolded within the larger picture of human-animal relationships in southwest Asia.

Ritual practice also contributes to variation in PPNB provisioning decisions [9,10]. The PPNB is characterized by increased archaeological visibility of ritual practice, especially rituals involving animals as food or symbols in events such as feasts, commemorations and funerals [11–15]. Provisioning these symbolic events was prescribed by ritual requirements for animals from certain species, ages, sexes, wild/tame statuses, or sizes [16,17] and thus differed from the economic concerns of more routine subsistence provisioning. Further complexity is added by the close interaction of ritual and economic practice that likely fed back into the perceptions and use of animals over the course of the domestication process [18–20].

This complexity is reflected in the archaeological record making it difficult to estimate the relative contributions of ritual and economic provisioning at Neolithic sites. Kfar HaHoresh (KHH) (ca. 10,600–8700 cal BP), Israel is a rare site in the Mediterranean Hills west of the Rift Valley that both coincided with much of the early period of animal management and served as a center for human burial and accompanying ritual practice rather than human habitation [21,22]. New faunal data from the 2010–2012 excavations at KHH thus add a much-needed PPNB database that can conveniently contribute to central questions on both the emergence and timing of animal management and ritual provisioning.

The KHH record is especially important and unique because it provides the longest continuous PPNB sequence in the Mediterranean Hills. The sequence includes much needed samples from the Early (EPPNB), Middle (MPPNB) and Late PPNB (LPPNB) (Table 1)—the former and latter being two crucial, yet under-represented cultural phases essential for investigating the evolution of animal management in the Mediterranean Hills. Currently, EPPNB faunal samples from the Mediterranean Hills are restricted to the site of Motza [23]. Even more important is the addition of the LPPNB sample, since only small LPPNB assemblages have been available to investigate continuity in the steps toward animal management and ritual practice up until now [5,24]. The picture of faunal use at KHH enables the investigation of steps toward animal management and ritual provisioning over the long-term at a single site.

**Table 1:** Southern Levant chronological framework based on dates from recent excavations in the region [25–28] and unpublished recent dates from KHH. *±50–100 years.

Period	Date (cal. BP)*
PPNA	11,650–10,600
EPPNB	10,600–10,000
MPPNB	10,000–9,500
LPPNB	9,500–8,700
FPPNB (PPNC)	8,700–8,350
PN	8,350–7,450

In this study of the 2010–2012 faunal assemblage from KHH, we evaluate whether human-animal relationships change across the E–LPPNB sequence. First, we seek evidence for emergent animal management in the relative abundance of wild game and domestic progenitor taxa in human diets. In particular, we examine how changes in the abundance of domestic progenitor taxa influence human hunting of wild ungulates, especially gazelle, which was intensively hunted by humans in the preceding Natufian and PPNA periods [29]. Next, we utilize classic indicators of herd management such as mortality profiles and body size change to investigate whether the domestic progenitor species at KHH were under some kind of human management and, if so, the nature of this control. This is achieved by investigating the relative frequencies, culling profiles and average body sizes of goat, pig and cattle populations over time. Finally, we consider the state of animal management in light of the ritual function of KHH to assess how the selection of animals compares with those from neighboring village settlements, such as Yiftah’el [24,30].

## Background

### Kfar HaHoresh (KHH)

KHH is the only known PPNB site in the southern Levant that served primarily as a ritual center, likely for surrounding habitation settlements [31]. Seventeen excavation seasons at the relatively small site (~1.5 acres) have uncovered unusual architectural features such as a monumental plastered podium/platform (the Locus 1604 complex), L-shaped and free-standing stone walls, and plastered surfaces, but no domestic structures [32]. Numerous human burials (n >85) with an array of treatments including primary and secondary, single and multiple interments and unique secondary inhumations such as a burial of half of a male skeleton and a possible depiction of an animal arranged from human long bones, attest to the funerary role of the site [11]. Iconic representations of ritual behavior tied to the south Levantine PPNB symbolic system have also been recovered, such as plastered human skulls with modeled facial features [33,34] and groupings of stone artifacts and figurines interpreted as male-centric items [11,22,32]. Material acquired from distant sources including malachite from the Dead Sea area, marine mollusks from the Mediterranean and Red seas and obsidian from southern Anatolia, demonstrate that KHH had exchange connections within the larger PPNB interaction sphere [22].

The function of KHH as a gathering place in the hills of the Galilee is supported by evidence for large-scale, collective rituals such as feasts [11,35,36]. Combined with the lack of arable land for agriculture on the hill slopes [21] and the absence of domestic structures despite extensive testing and long-term excavation, this sets KHH apart from PPNB habitation sites [37,38]. Furthermore, the site is located in the uppermost reaches of a valley surrounded entirely by steep hills. This sequestered location is hidden from the view of other sites, yet is located near a prominent landmark of the Lower Galilean landscape—the western ridge of the Nazareth Hills [21]. The apparent absence of domestic use, and its role as a ritual gathering place may have impacted how KHH was provisioned in comparison to neighboring PPNB settlements. This study lays out the economic background within which ritual practice at KHH occurred. Ultimately, this is essential for understanding how broader trends toward animal management in the southern Levant influenced decisions regarding the provisioning of animals at KHH.

### The Faunal Record: Intensive hunting prior to the PPNB

Significant evidence for an intensive hunting regime in the southern Levant is provided by the Epipaleolithic and PPNA record that directly precedes the PPNB in the Mediterranean Hills [29]. Increased inclusion of prey with lower payoffs and higher energetic costs of capture (lower ranked prey) indicates intensified hunting with the onset of a more sedentary lifestyle, especially during the Natufian period [29,39,40]. Intensive hunting is indicated by larger proportions of small-bodied gazelles in comparison to larger ungulates, high proportions of juvenile in comparison to adult gazelles, and increased abundances of small to large game [40,41], especially costly and more difficult to catch types. This pattern persists into the PPNA when low foraging efficiency is again indicated by high proportions of young gazelles [42] and high ratios of fast to slow small game, indicating continued intensive hunting as sedentary horticultural communities became more entrenched [43–45].

### Animal Domestication Research in the Southern Levant

The goat (*Capra* sp.) was the first herd animal to be managed in the southern Levant likely by the MPPNB (10,000–9,500 cal BP) [7,24], and thus postdates the earliest evidence for goat management in southeast Anatolia and the northern Levant by 900–1000 years (ca. 10,900–10,500 cal. BP) [7,46]. Given the greater time depth attributed to animal management in the north [47], it is important to establish how the southern Levant fits into the larger picture. Whether domestication occurred locally or managed animals were imported to this area is still a matter of debate. More data are needed to define local variation in the timing of onset and the pace of change across the region, particularly in the Mediterranean Hills.

The beginning of goat management in the southern Levant is typically identified by the frequency of goats in faunal assemblages. Although wild goats are native to the Levant, they were rarely hunted by Epipaleolithic or early Neolithic people [29,42,43,48]. Nevertheless, goat abundance rose substantially from less than 1% in the Epipaleolithic [40,42,48] to 10–55% by the MPPNB at Yiftah'el, Motza, Nahal Oren and Abu Gosh [5,49]. Recent data from Motza suggests that this increase may have begun even earlier during the EPPNB [50]. Despite variability in the scale of increase, goat abundance rises in the absence of climatic change [51]. It is becoming increasingly apparent that pig (*Sus scrofa*) and cattle (*Bos primigenius*) frequencies also began to increase slightly across the Mediterranean Hills as early as the EPPNB [30,52]. Since clear evidence for the full-fledged domestication of pig and cattle does not appear in the region until later [9,53], these changes have not been investigated as exhaustively as they have been for goats. Additionally, sheep have been identified at Late Natufian and Harifian sites in the Negev region [54] and appear in the Jordan Highlands ('Ain Ghazal) and northern Jordan Valley by the MPPNB, but do not appear in the Mediterranean Hills until the PPNC at Atlit Yam and Ashkelon [55]. Based on current data, it is generally agreed that the MPPNB marks the onset of animal management in the southern Levant [5,55].

### The Beginning of Animal Management in the Mediterranean Hills

**Fig 1:**
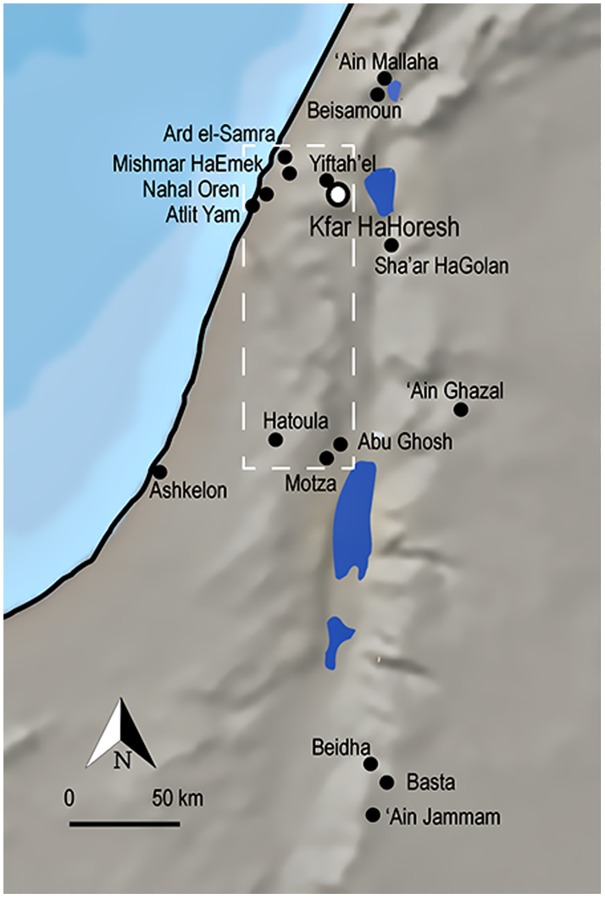
Map of south Levantine sites referenced in the text. The location of Kfar HaHoresh is marked with a hollow circle (32°42'20'' N 35°16'28'' E). Mediterranean Hills sites are outlined with a dashed line.

Although relative taxonomic abundance, demographic profiles and average body size are key for identifying early dates for animal management, variability in these measures and the lack of data in any given period renders a murky understanding of how this process unfolded on a regional scale. Because evidence for animal management varies significantly across ecological zones in the southern Levant [5,24,56], we narrow our comparisons to the Mediterranean Hills (Fig 1). Two competing hypotheses for the emergence of managed animals continue to be debated for this region. The first suggests that animals were domesticated locally, while the alternative states that managed animals were imported from the north. The importation argument has been supported by earlier dates for goat management in the north [46]. Supporters of this argument cite the early appearance of managed goats at sites within the Levantine corridor as evidence that domesticated animals first spread down the Jordan Valley then to the western and eastern regions of the Levant [57]. Managed goats are argued to have spread to neighboring regions within the southern Levant from there.

The autochthonous domestication scenario proposed for the Mediterranean Hills [24] harnesses increases in goat abundance at several sites as evidence for a local transition from hunting to herding across the PPNB–PPNC. Horwitz [56] argues that increased goat abundance compared to previous periods reflects incipient domestication. Horwitz also cites high juvenile kill-off in the goat assemblage from MPPNB Yiftah'el, Munhatta and Abu Gosh and LPPNB Tell Ramad as evidence for human control of goats. The gradual rate of change across the PPNB is cited as evidence for an *in situ* process of animal management in the Mediterranean Hills with changes in goat exploitation that differ in timing from other regions [24,58]. Horwitz interprets variability in goat frequencies, mortality profiles and average body-size as evidence that communities had varying degrees of participation in a wider, local autochthonous domestication process [24]. Increased frequencies of goats, in particular of adult females, observed in a new sample from Yiftah'el, support the argument that humans were exerting some control over animal populations from the MPPNB and perhaps as early as the EPPNB [30]. Clearly, more faunal data are needed to understand how these processes developed over time. With its multiple PPNB occupation phases, KHH provides exactly such a dataset.

## Methods

This study investigates whether animal management emerges during the PPNB at KHH and if so, when. Next, it highlights how human-animal interactions developed over the course of the PPNB to address long-term change at the site level. We investigate these changes by laying out expectations for animal management in relative taxonomic abundance indices, ungulate mortality profiles and body-size indices and then compare them with data from KHH. The results from KHH are also compared to other PPNB period sites in the region including Yiftah'el, Motza and Abu Gosh to reassess the timing and scale of early animal management in the region.

### Expectations for Animal Management

#### Wild game hunting

The emergence of animal management at KHH is investigated by examining changes in hunting intensity and the relative frequency of domestic progenitor taxa in the faunal assemblage. Animal management and ultimately, domestication reflect increasing human control over animal movement, reproduction and diet [7]. Limiting the movement of high-ranked ungulate taxa with characteristics amenable to human control, ultimately increases their encounter rate and reduces search costs [1]. Although the control of animals can be costly, confinement and ultimately reproductive control increases their population density and eventually their rate of population growth [59]. As human control over and accessibility to high-ranked domestic taxa increases, these taxa should increase in faunal assemblages. Because they are highly ranked, a narrowing of the diet will ultimately reduce hunting intensity and increase foraging efficiency [60,61]. Animal management is thus expected to result in reduced taxonomic diversity [62], higher proportions of domestic progenitor taxa in comparison to wild game [45,63] and associated decreases in lower-ranked game including smaller-bodied ungulates, such as gazelles, small game taxa (tortoises, hares and birds) and juveniles in comparison to adult gazelles [29,40]. Taxonomic diversity is calculated with PAST software using Simpson's D [64]. The relative abundance of juvenile and adult gazelles is investigated using classic measures of ungulate mortality including tooth wear and eruption sequences and bone epiphyseal fusion [65–67].

#### Human interaction with domestic progenitor populations

Animal use goals are expected to shift over the transition from hunting to herding as the conception of animals changes from resources to property [68]. Further human control over animal reproduction is expected to maximize animal resources to meet herd security goals, starting with the optimization of meat production [67]. Small-scale animal management strategies are expected to promote herd security by culling the less essential male animals early in life, and maximizing the number of productive adult females needed to maintain the herd [69–71]. Measures of population mortality of domestic progenitor taxa are used to investigate whether there was a shift from an immediate to a delayed returns strategy of animal use [44,72]. Mortality profiles are created based on bone fusion for goats [73], cattle [74] and pigs [75]. Samples of teeth from these taxa were too small to crosscheck with wear and eruption data.

Management frequently results in a decline in the average body size of animal taxa [76]. Initially, this decline is at least partially related to the change in demographic structure mentioned above—a higher adult female to adult male ratio [71]. Since females are smaller bodied than males on average, the average size of the population should decline when juvenile males are preferentially culled [71]. The Logarithmic Size Index (LSI) [77] is used to investigate the average body size of domestic progenitor taxa from KHH over the course of the PPNB. The LSI compares the size of individual elements to a standard animal and combines measurements from different elements to increase sample size. Here we use the Uerpmann and Uerpmann [78] standard animal for goat and Grigson’s [74] standard animal for cattle. Wild boar sample sizes were too small for LSI analysis. The skewing of the LSI distribution is used to investigate sex ratio—positive or negative skewing suggests more small-bodied (likely females) or large-bodied animals in the population, while no skewing indicates a normalized distribution that reflects a living population structure [79].

## Materials

The faunal sample studied here includes the material recovered during the most recent excavation seasons at KHH (2010–2012). The sample derives from the most securely dated contexts and loci spanning Early, Middle and Late PPNB (EPPNB, MPPNB, LPPNB) periods. Chronological assessments were made based on stratigraphic correlations and radiocarbon dates [80]. The density of fauna and other material at KHH increased significantly through time, likely due to increased occupation intensity [21]. The faunal sample originates from loci in the northern part of the site. Sampled loci range widely in function and include densely packed middens with embedded stone-lined garbage deposits, deposits sandwiched between layers of plaster applied on architectural features, graves, hearths, caches of finds such as flint, and pits associated with the on-site production of plaster, lithics and food. Thirty-nine percent (NISP = 16,905) of the recovered fragments were identified to element and to animal body-size category or more specific taxonomic group and attest to the good quality of preservation at the site. Specimens were identified using the comparative vertebrate collections in the National Natural History Collections of the Hebrew University, Jerusalem. Statistical analysis was performed with PAST software [64].

### Ethics Statement

Excavations were directed by one of the authors (A.N. Goring-Morris) from the Hebrew University, Jerusalem on behalf of the Israeli Antiquities Authority (excavation licenses G-29/2010, G-43/2011, G-60/2012). The zooarchaeological specimens (#1–16,905) can be accessed at the National Natural History Collections of the Hebrew University, Jerusalem.

## Results

### Hunting Intensity

#### Taxonomic diversity and relative abundance

**Fig 2:**
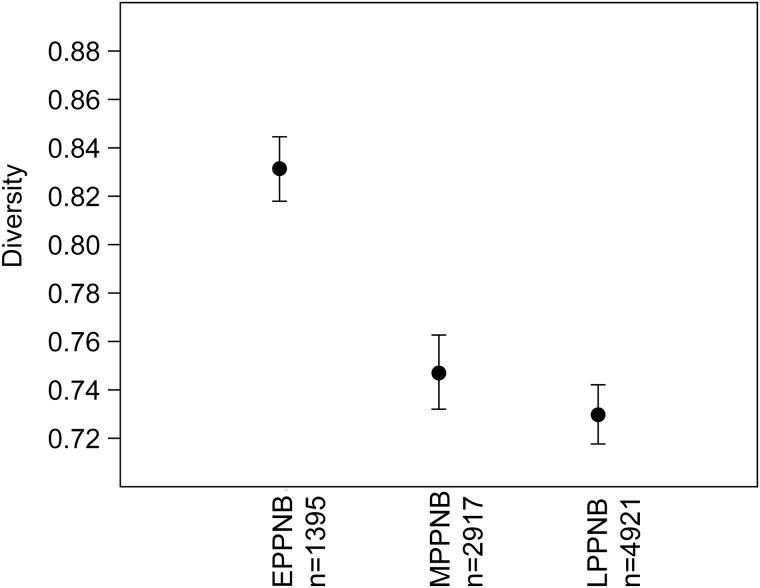
KHH Simpson's diversity index by site occupation phase.

**Fig 3:**
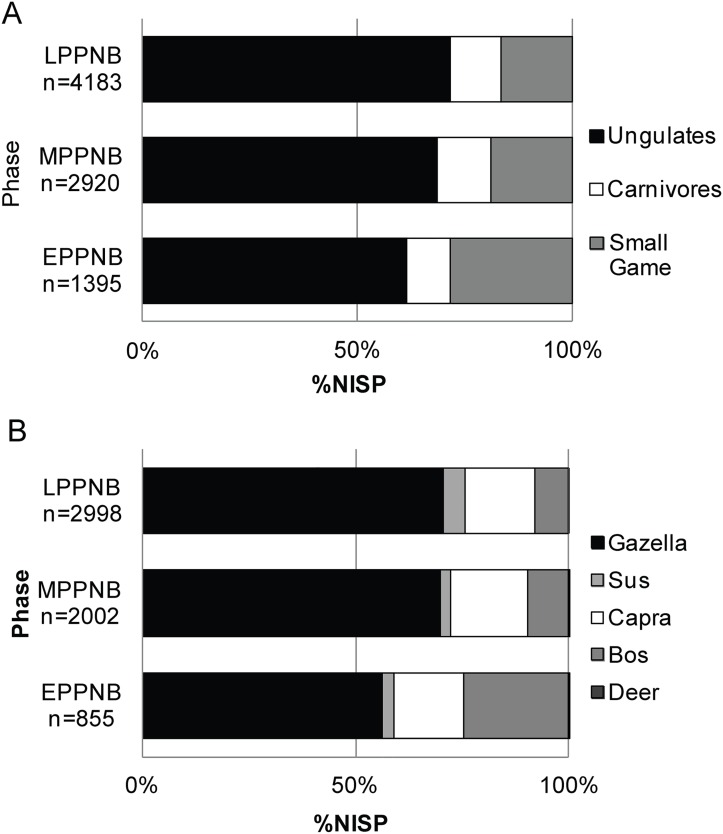
KHH relative abundance of (A) broad taxonomic groups and (B) relative ungulate species abundance based on %NISP per taxa (S1 Table).

At KHH, species diversity decreased significantly between the EPPNB and later periods from 0.83 to 0.73 (Simpson's D, t-test of similarity: E–M t = -8.11, p< .01, E–L t = -11, p< .01, M–L t = -1.7, p = .08) (Fig 2). This shift reflects the increasing abundance of high-ranked ungulates in lieu of lower-ranked small game and carnivores over time (Fig 3A). These results fit a drop in taxonomic richness observed by Horwitz [62] across the southern Levant from the PPNA to the MPPNB (Menhenick Index, D_mn_ = 1.3 to 0.4). Ungulates dominate the KHH assemblage in all periods, but increase from 61 to 72% over time (Fig 3A). This corresponds to a decline in small game abundance from 28 to 17% from the E–LPPNB. Carnivore abundance fluctuates from 10 to 12% between the E–LPPNB.

Gazelle, the dominant taxon consumed by humans in the southern Levant throughout the Epipaleolithic (~83–98%) [40,42,45] and the PPNA (73–88%) [42], is less common in all PPNB phases at KHH (Fig 3B) than in preceding periods in the Mediterranean Hills. Again, this fits with similar trends across the region [43,56], but this is where the similarity ends. Unlike the general decline in gazelle abundance across the Levant that continues into the PPNB, the relative abundance of gazelles out of all ungulates increases significantly at KHH between the EPPNB (56%) and MPPNB (70%) and then stabilizes in the LPPNB (70%) at KHH (Fig 3B).

#### Mortality profiles for gazelle

**Fig 4:**
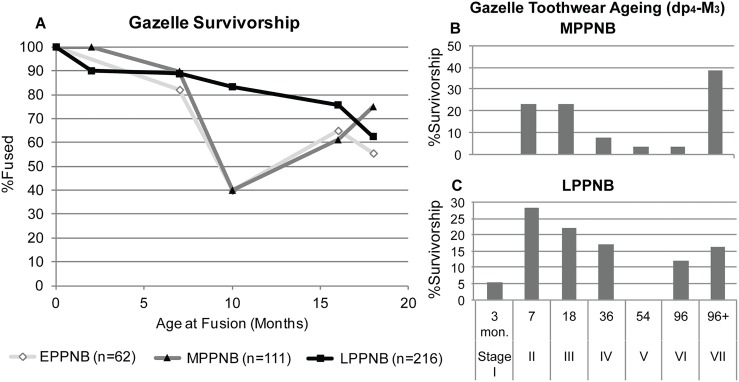
KHH gazelle survivorship curves (A) based on percentages of fused bone elements (see S2 Table for MNE values) and age stages based on gazelle toothwear (following [65]) for the (B) MPPNB (n = 13) and (C) LPPNB (n = 16) phases. Survivorship curve changes are not significant based on Kolmogorov-Smirnov test (K-S) (E to M p = .75, M to L p = .69, E to L p = .25).

The abundance of juvenile gazelle in all PPNB phases represented at KHH (23–39%) is less than at earlier Natufian (up to 45%) and PPNA (59%) sites in the region [40,44]. Nevertheless, the ages of gazelles represented at KHH changed little over time. Analysis of gazelle survivorship based on bone fusion indicates that the kill-off of juvenile gazelles less than 18 months of age remained stable at 35% and 39% in the E–MPPNB and then decreased to 24% in the LPPNB (Fig 4A). The tooth wear results from KHH are similar and indicate that juvenile gazelle survivorship changes, but not significantly (K-S test p = .88) from the MPPNB (23%)(Fig 4B) to the LPPNB (34%)(Fig 4C). There are slightly fewer old and prime-aged animals in the LPPNB. Gazelle tooth sample sizes from the EPPNB were too small for analysis. In all cases, juvenile representation is similar to or less than the 33% of young animals present in stable modern gazelle populations [81]. Thus, gazelle demographics at KHH resemble a natural population structure, and do not reflect prime-dominated hunting as expected if hunting intensity had continued to decline. The KHH gazelle data is similar to gazelles from EPPNB Motza (28% kill-off before 18 months of age) [23] and the recent findings from MPPNB Yiftah'el (26% kill-off before 18 months of age) but differs in abundance from later PPNB sites in the region [30,50].

### Indicators of Animal Management

#### Relative ungulate abundance

Together, the three domestic progenitor taxa are represented in much higher frequencies in all PPNB layers at KHH than in earlier Epipaleolithic and Neolithic sites in the Mediterranean Hills (Fig 3B). In particular, the abundance of *Capra* (16–18%) in relation to other ungulates at KHH is significantly higher in all PPNB periods than at Epipaleolithic (<1%) [40,42,48], PPNA (1–3%) [82–84], and EPPNB (3%) [23] sites in the region. The percentage of goats at EPPNB KHH (16.3%) is higher than expected—and is most similar to MPPNB sites in the Mediterranean Hills, including Yiftah'el (Areas C&D)(16.6%), Motza (15.7%), and PPNB Ard el-Samra (19%), albeit significantly lower than MPPNB Abu Gosh (54%), which stands apart from other sites in the region [23,24,85,86].

**Fig 5:**
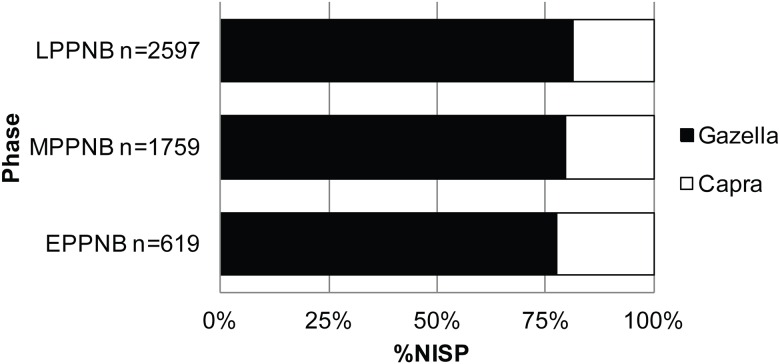
KHH *Capra* (white) to *Gazella* (black) ratio over time (%NISP).

Also, unlike the region-wide trend, the abundance of *Capra* remains steady over time at KHH (*Capra*:16–18%). The same is true of the ratio of goat to gazelles, which is typically used to track the onset of goat management (gazelle:goat EPPNB 3.4:1; MPPNB 3.8:1; LPPNB 4.3:1) (Fig 5). Although other faunal assemblages from the Mediterranean Hills are dominated by gazelle during the PPNB [55], the gazelle to goat ratio decreases over time at other sites. This is especially clear from the E–MPPNB at Motza (20:1 to 4:1) [23] and at Yiftah'el (MPPNB 3.7:1; Final PPNB/PPNC 0.1:1 Areas A, B, C) [24].

The increase in gazelle at KHH coincides with a decrease in *Bos* over time, especially from the EPPNB (25%) to the MPPNB (8%) (Fig 3B). Like *Capra*, the relative abundance of *Sus* remains low and steady over time (*Sus*: 3–5%). No sheep were identified in the assemblage.

The relative abundance of cattle compared to other ungulates in the EPPNB (25%) at KHH is higher than many other sites in the area, including EPPNB (3%) and MPPNB Motza (4%), Nahal Oren (2.4%), Yiftah'el Areas C&D (10%), and some MPPNB layers at Abu Gosh (3–19%) [23,24,85,87,88]. In contrast, *Sus* abundance at KHH is similar to earlier PPNA sites in the area, such as Hatoula (2%) and Nahal Oren (3.5%) and is lower than MPPNB sites in the area including Yiftah'el Areas C&D (10%), Abu Gosh (10–11%), and Motza (13%), but not MPPNB Nahal Oren (4.6%) [23,24,83,85,87,88].

#### Mortality profiles for goat, cattle, and pig

**Fig 6:**
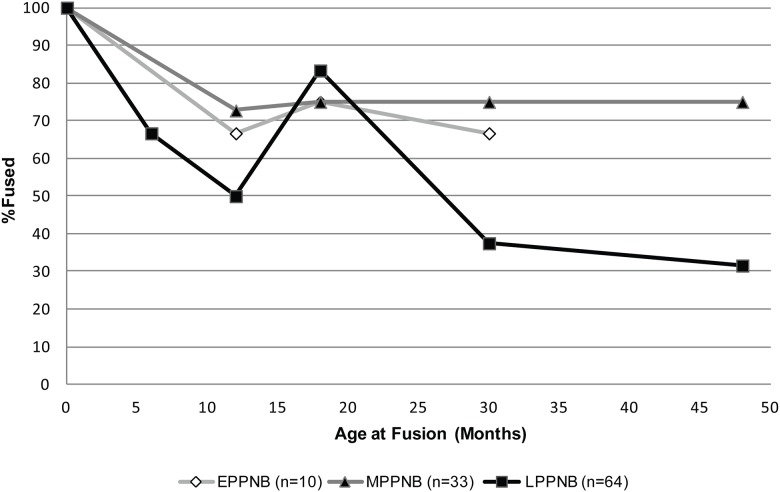
KHH *Capra* Survivorship by PPNB phase. There is a significant difference between the M and LPPNB profiles (K-S test p = .036). Elements with fewer than three total fused and unfused specimens were removed (see S3 Table), including proximal humeri from all periods. No data for age stage 6 (>48 months).

The mortality profiles based on the bone fusion of domestic progenitor taxa from KHH show some change over the course of the PPNB. *Capra* mortality drops only slightly from the E–MPPNB, but the survivorship of animals less than 48 months of age drops significantly from 75% to 32% between the M–LPPNB (Fig 6). This is lower than the survivorship of a modern wild population of *C*. *aegagrus* from Pakistan (60% to 48 months of age) [89]. *Capra* demographics at KHH are consistent with PPNB sites in the region, such as MPPNB Abu Gosh (31% survivorship at 39 months) [85] and Final LPPNB/PPNC Yiftah'el (33% survivorship at 42 months) [24] that have been interpreted as early managed populations.

**Fig 7:**
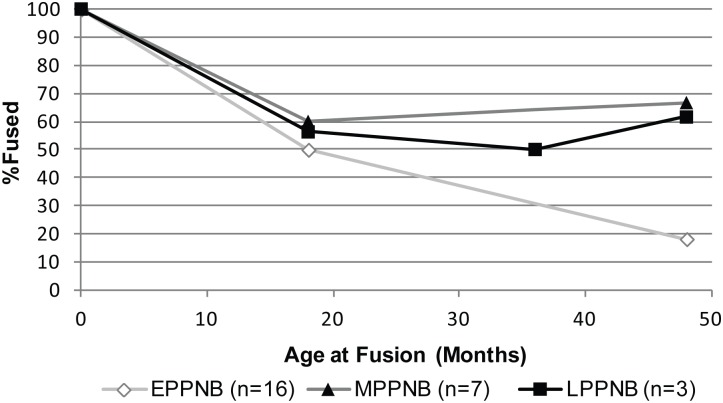
*Bos* survivorship curve based on fusion data. Elements with total MNE < 3 were not plotted (see S4 Table). There is a significant difference (K-S test) between the Early and Middle PPNB profiles (p = .03) and Early and Late PPNB profiles (p = .031), but no significant difference between the Middle and Late PPNB profiles (p = .31).

In contrast, there is a significant increase in the survivorship of cattle less than 48 months of age at KHH from 18% in the EPPNB to 60–66% by the M–LPPNB (Fig 7). Although comparisons are limited by very small sample sizes, a rise in the survivorship of adult cattle to 48 months of age was also noted at Yiftah’el from the MPPNB (40–75%) (Areas C and D; n = 9) to the FPPNB/PPNC (100% by 36 months of age) (Areas A and B; n = 6) [24] and at other PPNC sites in the region [52]. Sample sizes at Yiftah'el are not large enough to be as certain of whether a shift in survivorship occurred over time.

**Fig 8:**
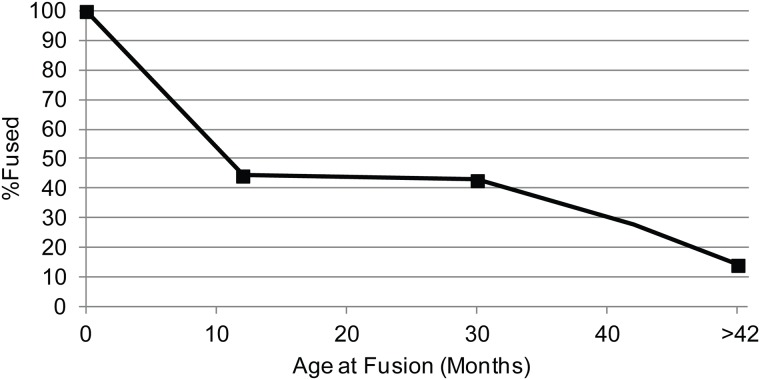
Survivorship curve for *Sus* (n = 23) at LPPNB KHH based on age at bone fusion [75]. See S5 Table for MNE values. Elements with MNE< 3 were not plotted.

Of the *Sus* assemblages, only the LPPNB sample is large enough for mortality analysis based on fusion data (n = 23). This assemblage provides the first *Sus* mortality data from the LPPNB in the Mediterranean Hills. It shows that most of the population was culled before adulthood (Fig 8). Forty-four percent survived to one year of age. This is less than the survivorship to one year of age in modern wild *Sus* populations in Spain in mountainous (58.6%) and riverine habitats (75%) [90]. Survivorship of *Sus* to one year of age (44%) is also lower at LPPNB KHH than at MPPNB Abu Gosh (98%) [85] and Yiftah'el (60%) [24], and at PPNC (80%) Sha'ar HaGolan [91].

#### Average body-size of domestic progenitor taxa

**Fig 9:**
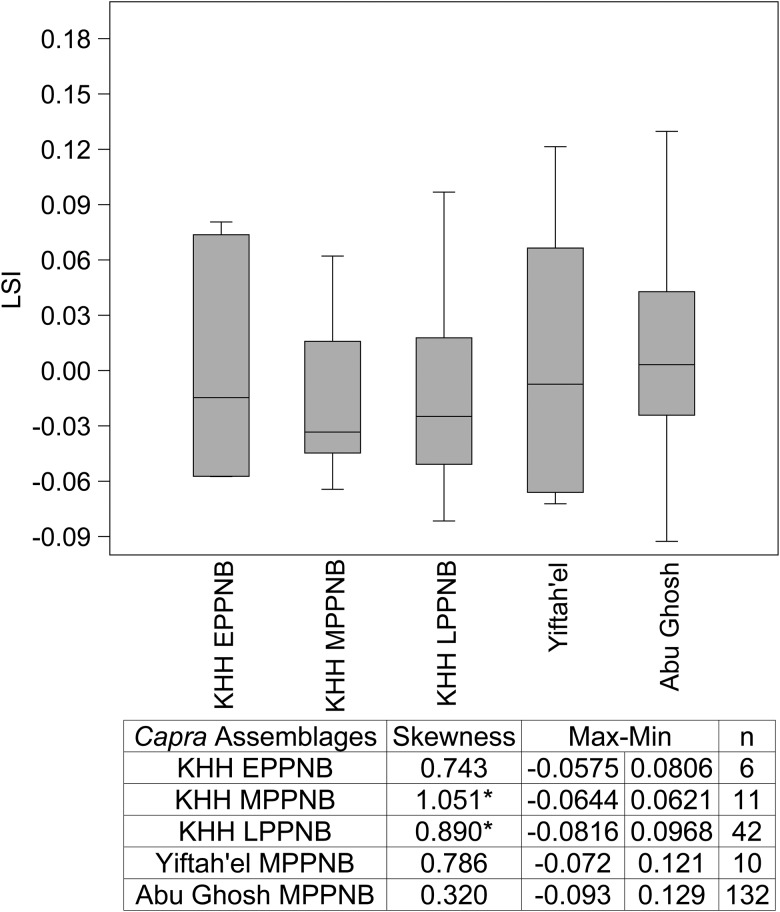
Box plots of LSI value medians, inter-quartile ranges, minimums and maximums for KHH *Capra* compared to Yiftah'el [24] and Abu Gosh [87]. Only one measurement included per specimen, only breadth/depth measurements were used (S6 Table) following [77]. Yiftah'el sample from Areas C and D. *Outside 90% range for normal symmetric population given the sample sizes [93].

**Fig 10:**
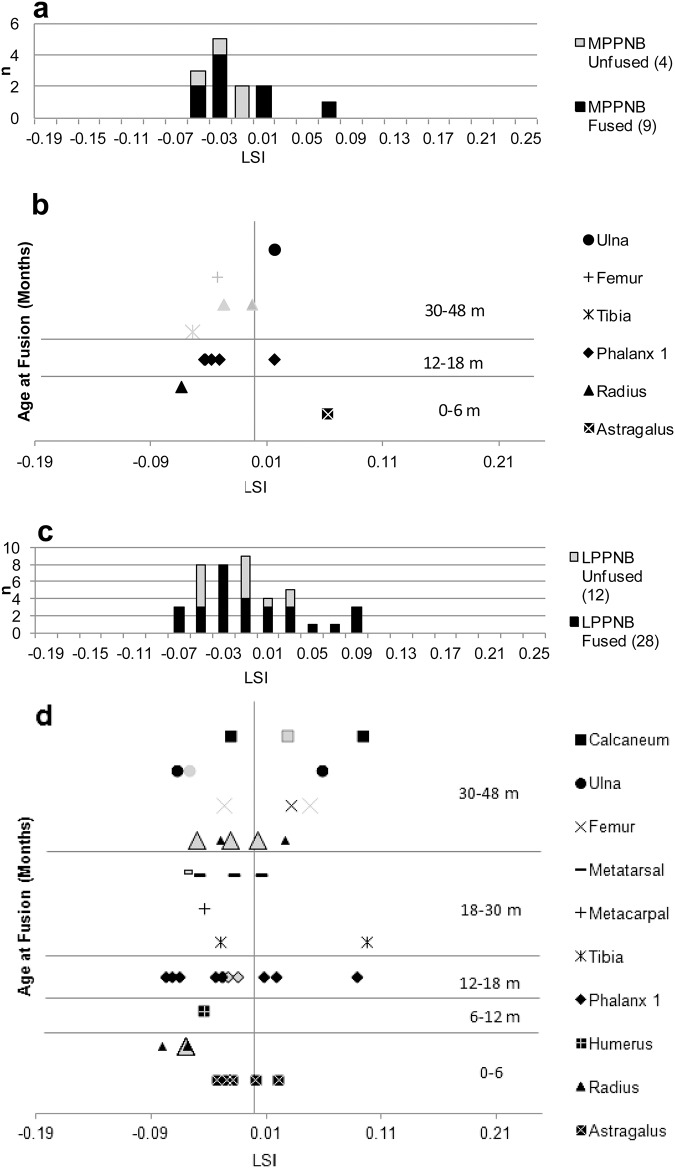
*Capra* LSI values from KHH based on comparison to standard [78]. **(A) KHH MPPNB LSI and (B) MPPNB single-element LSI values in order of age at fusion. (C) KHH LPPNB *Capra* LSI and (D) LPPNB single-element LSI values in order of age at fusion**. Measurements listed in S6 Table. Unfused elements shown in grey, fused elements shown in black.

At KHH, the average body-size of goats decreases slightly from the E–MPPNB (-0.004 to -0.019) (Fig 9, S6 Table). This decline is not statistically significant (t-test for similarity of means, p = .55). Notably, LSI distributions of fused elements are skewed substantially to the right in all periods (Early 0.7, Middle 1.05, Late 0.8) (Figs 9 and 10A–10D), suggesting a high proportion of smaller-bodied females in the population. In the LPPNB assemblage, an increase in young male caprine kill-off is suggested by the fact that 73% of unfused elements have larger LSI values than fused specimens of the same element (Fig 10C and 10D)—the unfused elements are likely males, which can exceed females from the same population in size as early as 12 months of age [92].

**Fig 11:**
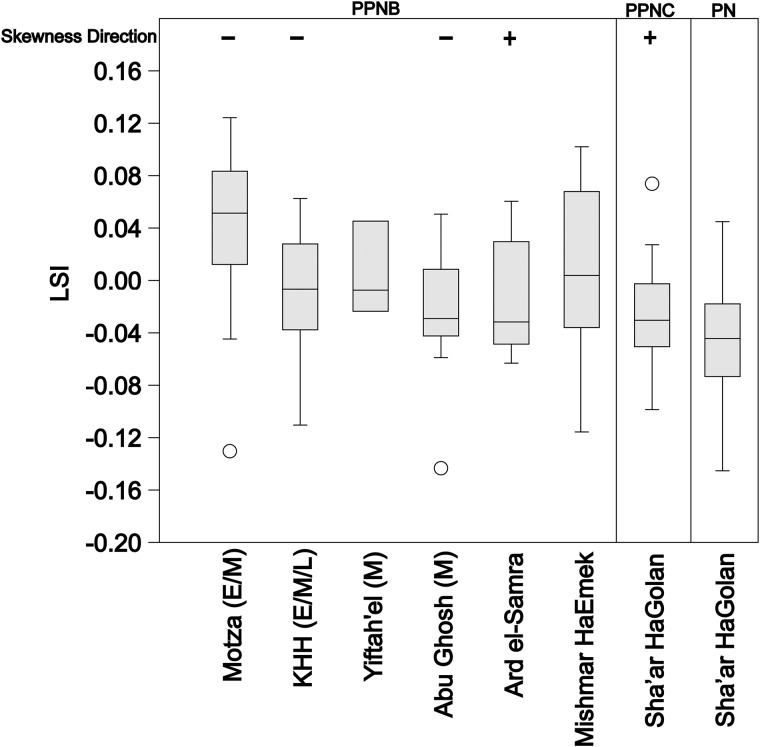
*Bos* LSI box plots (±1 SD shown) from KHH (S7 Table) and other Neolithic sites in the Mediterranean zone and Jordan Valley over time. Box plots of *Bos* LSI value medians, inter-quartile ranges, maximum and minimum values with outliers (open circles) based on comparison to the standard female from Grigson [74]. Sites listed with PPNB phase when possible (E = Early, M = Middle, L = Late). KHH measurements (n = 24) include four previously published values [96]. Sources of other measurements: Motza (n = 17) [94], Yiftah'el (n = 4) [24], Abu Gosh (n = 14) [87], Ard el-Samra (n = 8) [86]. Mishmar HaEmek PPNB (n = 28), Sha'ar HaGolan PPNC (n = 18), PN (n = 44) [91]. Direction of skewness noted for right (+) and left (-) skewed assemblages when skewness is substantial (≤-0.5 or ≥0.5).

LSI data for south Levantine goat populations around the time of the transition to agriculture are spotty, but substantial enough for comparisons to earlier wild and later managed populations. The average LSI for the KHH goats from the M–LPPNB periods (Mean LSI -0.019 to -0.015) is smaller than wild populations from the Natufian period (Eynan, 0.034), but significantly larger than the fully domesticated herds from the western highlands of Jordan in the Yarmoukian period ('Ain Ghazal, -0.044) [5]. The same is true of all other measured populations of MPPNB goats from the Mediterranean southern Levant including Abu Gosh (0.008) [87], and Yiftah’el (0.004) [24]. Nevertheless, there is variation among the Mediterranean PPNB populations. The average LSI values from KHH are smaller than those from MPPNB Yiftah'el [24] and Abu Gosh [85], even when unfused specimens are removed from the analyses. Results of the new analysis of fauna from Areas G and I at MPPNB Yiftah'el [30] include unfused specimens and are smaller on average (-0.011) than specimens from Horwitz’s [24] MPPNB sample from the site. They are more positively skewed (-0.1) toward the smaller end of the population than *Capra* from EPPNB Motza (-0.7), despite prime-dominated age profiles [94]. The KHH distributions are more skewed to the right (positive) than the Abu Gosh distribution, which is skewed only slightly to the right. Larger average body size at Abu Gosh may relate to the inclusion of *C*. *ibex* measurements in the LSI data [95], but the LSI distribution is positively skewed, and likely indicates a slight female bias. The *Bos* LSI distribution from KHH combines measurements from all PPNB phases to maximize sample size and includes many measurable bones that could be dated only to the PPNB. *Bos* from KHH are similar in size to those from other south Levantine PPNB sites (Fig 11). Although geographic variation likely influenced *Bos* body-size, there is a significant decline from the EPPNB to the PN in sites from the Mediterranean Hills and Jordan Valley. Cattle from KHH are significantly smaller than those from E–MPPNB Motza [94] (Mann-Whitney Pairwise test for similarity of means, p < .005), but significantly larger than those from the PN phase from Sha'ar HaGolan [91] (p < .001) (Fig 10). The decline in *Bos* body-size is most pronounced between the FPPNB and the PN at Sha'ar HaGolan [91]. *Bos* LSI distributions from KHH are negatively skewed and likely favor males, much like distributions from Motza and Abu Gosh, but differ from the positively skewed distribution at Ard el-Samra [86] and PPNC Sha'ar HaGolan [91] and the normal distributions from Yiftah'el (Areas C&D) [24] and Mishmar HaEmek [91].

## Discussion

### Hunting Intensity at KHH

In comparison to Epipaleolithic and PPNA sites in the Mediterranean Hills, the fauna from KHH reveal a narrowing of dietary breadth and an increase in foraging efficiency over time [60,61,97]. This fits a region-wide trend reflecting a trade-off between small game and domestic progenitor species from the Natufian to the PPNB [43,56]. In addition, there is a clear increase in the abundance of adult gazelle compared to the juvenile-dominated assemblages of the PPNA and Natufian [40,42]. At KHH, both the decline in taxonomic diversity and the decrease in small game abundance indicate the continuation of this trend toward increased foraging efficiency through the PPNB. Nevertheless, high abundances of gazelle across the PPNB occupation at KHH signify the opposite trend. The increase in the lowest-ranked gazelles at the expense of the highest-ranked cattle indicates a decline in ungulate foraging efficiency at KHH over time.

Thus, some changes in wild game proportions at KHH follow expectations for an economy that includes nascent animal management and support a trajectory toward less intensive hunting from the Natufian and PPNA through the end of the PPNB, while others do not. Reduced hunting intensity corresponds to the increase in domestic progenitor taxa in the ungulate fraction of the assemblages and thus fits expectations of emergent management of some ungulate taxa according to regional faunal trends [62,98]. Although the relative abundance of domestic progenitor species in the PPNB is significantly greater than assemblages that came before, the steady emphasis on gazelles throughout the PPNB at KHH reveals a leveling off of this trajectory at least in the ungulate component. Continued reliance on gazelles during the PPNB at KHH differs from the steady increase in domestic progenitor species seen across the southern Levant more generally from the MPPNB through the PN, which has been associated with greater control of these taxa over time [43,55].

### Animal Management at KHH

Increasing frequencies of *Capra* are commonly cited as markers for the beginning of caprine management at PPNB sites in the Levant [5,24,42,99]. KHH fits the established regional pattern of increased *Capra* abundance compared to the Epipaleolithic and PPNA. Interestingly, however, the rise occurs earlier than expected in the EPPNB. The proportions for *Capra* in all phases at KHH are most similar to MPPNB sites in the region (16–19%) [23,24,86], with the exception of Abu Gosh, which is higher [85]. This suggests that human control over goats may have begun earlier than previously established for the Mediterranean Hills (see also [30,94]). Nevertheless, despite the initial increase, goat abundance remains stable over time at KHH and thus, diverges from the trajectory of increase that typifies later LPPNB sites in adjacent regions such as Beisamoun (53%) [100].

Other features of the KHH *Capra* populations also resonate with those detected at other Mediterranean Hills sites by the MPPNB. The KHH *Capra* LSI body-size data are similar to those from neighboring PPNB sites suggesting that a region-wide shift in goat body size occurred over time. These goat populations were slightly smaller than the Natufian wild assemblage from Eynan, but not as small as later PN assemblages. Additionally, by the LPPNB goat mortality profiles at KHH approach 30% survivorship by 36 months of age (37% survive to 30 months at KHH based on bone fusion categories), as expected for modeled caprine populations managed for meat [67]. Although *Capra* survivorship varies across the region, KHH is most similar to sites with the lowest juvenile survivorship that have been interpreted as early managed populations (MPPNB Abu Gosh and Final LPPNB/PPNC Yiftah'el) [24,85].

Evidence for small-scale size diminution and a younger average age of culling in the EPPNB and MPPNB at KHH compared to earlier periods illuminates very early signs of a shifting relationship between humans and goats during the period leading up to animal domestication in the region. These changes are not sufficient to argue for full-fledged animal management, but they do suggest a shift in the relationship between goats and humans that is similar to other Mediterranean Hills sites. Positively-skewed LSI distributions at MPPNB Yiftah'el and KHH suggest that humans might have first gained control over smaller females that were likely easier to control than males. This very early stage of human control likely precluded directed reproduction and selective culling, but aimed to cut costs by improving access to high-ranked animals and reducing search time. Ultimately, this was followed by targeted culling of younger, likely male animals once they neared full body-size, just prior to sexual maturity. Constraining the movement of wild goats would have decreased search and capture costs and made it increasingly worthwhile to harvest goats rather than gazelles at Mediterranean Hills sites. What makes KHH so interesting is its continued focus on gazelle hunting despite increasing availability of lightly managed goats in the Mediterranean Hills. In this sense, the trajectory of change from the MPPNB onward at KHH diverges from the rest of the region.

The relative abundance of *Bos* in all PPNB phases at KHH, especially in the EPPNB phase, is notably higher than in preceding periods elsewhere in the southern Levant. Interestingly, *Bos* abundance peaks in the EPPNB and then declines over time counter to expectations for cattle management. In contrast, the abundance of *Sus* at KHH is not significantly different from earlier periods in the region and increases only slightly over time. Like *Capra*, the abundance of large domestic progenitor taxa at KHH from the MPPNB onward do not significantly increase as they do at other sites such as Yiftah'el [30]. Archaeological contexts suggest that species abundance is likely an unreliable marker for *Bos* management at KHH, as cattle were mainly recovered from concentrated EPPNB deposits probably related to feasting or other special activities [36]. Thus, *Bos* abundance, at least in the EPPNB, best reflects specific, short-term activities.

Comparison of *Bos* data from KHH to that from other sites in the region highlights a decline in average body size across the PPNB, indicating that KHH follows a similar pattern to sites in the Mediterranean Hills and in the Jordan Valley. The sample of *Bos* measurements from PPNB sites is very limited, but *Bos* LSI values are smaller on average than those from E–MPPNB Motza, suggesting that the decline in average cattle size may have begun earlier than the PPNC [49,52] or PN [101] as previously believed. This new data for *Bos* parallels and is coeval with the trend toward goat diminution and could similarly reflect the impact of increased human control over these domestic progenitor taxa in the region. Nevertheless, sex biases in cattle populations suggested by skewness of the LSI values vary widely on a regional scale and unlike goats, do not exhibit the female-biased adult populations characteristic of managed herds. This may be at least partially attributable to the small sample of *Bos* bones [52]. The increased survivorship of juvenile cattle also does not fit expectations for increased human control of *Bos* populations at KHH. This differs from the more juvenile, female-biased, or smaller cattle populations at LPPNB sites in Jordan (Basta, Beidha, 'Ain Ghazal) that have been interpreted as likely managed herds [12,52,55,102].

Accordingly, demographic evidence does not support *Bos* management at KHH. Instead, changes in the *Bos* age structure likely reflect a reduction in hunting pressure on wild cattle, similar to that shown by gazelles at the site. The alleviation of hunting pressure on *Bos* populations suggested by increased juvenile survivorship at KHH may reflect a trade-off resulting from intensified use of other domestic progenitor taxa, as is suggested by the age profiles of gazelles. In the Jordan Valley, a similar drop in the kill-off of juvenile cattle occurs later between the PPNC and the PN at Sha'ar HaGolan [91]. Marom and Bar-Oz [91] interpret this as evidence for the beginning of conservation of cattle herds in the PN in response to overhunting in the PPNC. Thus, even though the *Bos* data from KHH fit the regional body size pattern, the relative abundance and demographics of cattle populations at the site better fit a wild population rebounding from hunting pressure that was selected for specialized use in specific ritual practices (see below).

Finally, although small sample sizes prevent tracking of demographic change in *Sus* populations over time, the survivorship of juvenile pigs from LPPNB KHH is similar to survivorship at PN Sha'ar HaGolan [91], where it has been treated as evidence for domestic animals at the site. The survivorship of juvenile *Sus* at KHH is also lower than at earlier PPNB sites [24,75,85]. Still, diverse potential pig management strategies [103] are known to cause significant demographic variability in domestic populations, leading to equifinality in *Sus* demographic profiles. Hunting is also expected to produce higher proportions of juveniles than in other progenitor taxa since more young *Sus* are naturally available due to high rates of fecundity [104]. Thus, the *Sus* data is suggestive and hints at a small degree of human control, but this pattern is equivocal compared to the *Capra* data. Evidence of *Sus* management demands further investigation in the Mediterranean Hills region. Despite the high rate of juvenile culling, *Sus* abundances at KHH remain steadily lower than at other PPNB sites, and differ from regional trends reflecting increased use of *Sus* across the course of the PPNB.

### Provisioning a Ritual Site

In terms of the overall decline in hunting intensity, changes in the demographic profiles and body size of goats, and perhaps the demographics of pigs, the faunal populations that were drawn from to provision KHH resemble those used to provision surrounding PPNB sites in the region. In this sense, the fauna reflects region-wide trends of emergent early animal management, at least for goats by the MPPNB. Nevertheless, despite these similarities, the relative abundance of taxa selected to provision KHH differs significantly from the selection of taxa and the trajectory of change in species abundance at other PPNB sites—specifically, wild taxa continue to be emphasized at KHH even as animal management becomes more entrenched throughout the southern Levant. In particular, wild cattle are common at EPPNB KHH, gazelles abound throughout the sequence, and domestic progenitor taxa remain stable from the MPPNB onward. Residents of KHH clearly selected ungulates (early managed goats and possibly pigs, wild gazelle and wild cattle) from the same pool of animals accessed by residents of PPN sites in the surrounding area, but they made different choices about the relative quantities of these animals when provisioning the site.

This difference likely reflects the nature of activities that were performed at KHH compared to other sites in the region. As reviewed above, KHH lacks domestic areas and provides clear evidence for special ritual activities, often associated with burial events. Animal selection at KHH likely reflects provisioning decisions related more to the social and ideological goals of ritual practice such as feasting, than those of other sites [11,36]. The more mundane processes involved in the nascent management of *Capra* and possibly *Sus* populations likely occurred near more permanent neighboring settlements than at this isolated mortuary site.

The preference for wild animals, in particular gazelles, over domestic progenitor species is maintained at KHH across the PPNB. Although gazelles are more common in Mediterranean Hills assemblages [5,55], the stability of gazelle hunting at KHH strongly contrasts with the broader pattern of intensified animal management both in the Mediterranean Hills and throughout the southern Levant and greater Southwest Asia once animal management begins. A continued preference for wild gazelles despite the focus on increasingly managed animals at contemporaneous sites, suggests an inversion of the norms of food selection—a common feature of ritual practice [19,105]. Wild gazelle preference at KHH is undoubtedly related to its important role in PPN ritual practices, especially once the process of animal management began. The importance of gazelle in ritual practice is attested by unusual deposits at PPNB sites including a headless gazelle carcass burial associated with a plastered human skull at KHH [11], a pair of burned gazelle horns in a human grave at Motza [25], a gazelle skull placed in a wall niche at LPPNB 'Ain Jammam [106], and several gazelle horn pairs recovered on the floor of a building and, in the courtyard outside, an articulated gazelle carcass with burned feet at LPPNB 'Ain Ghazal [107].

Additionally, persistent use of wild gazelles as the primary source of meat provisions at KHH may have solidified traditions of feeding site visitors/congregants by producing continuity with past ritual performances. Similarly, PPNB mortuary practices that were centered around ancestors reaffirm connections with the past, such as those involving the reopening of graves and plastering of human skulls with sculpted facial features [108,109]. The continuity of gazelle hunting over time, despite the abundance of goats available at KHH by the EPPNB, reveals that the social benefits of using this wild species at the site exceeded the caloric benefits of using controlled taxa.

Abundant cattle in the EPPNB is clearly also related to the role of this wild species in ritual practices at the site and elsewhere [11,36,109,110]. Cattle were recovered from more structured deposits than other taxa in the 2010–2012 faunal sample at KHH. These included an EPPNB pit (Locus 2268) with highly concentrated *Bos* remains associated with the monumental Locus 1604 complex podium and M–LPPNB midden deposits [36]. These deposits comprise the majority of the *Bos* assemblage and inflate the abundance of *Bos* during the EPPNB phase. Additionally, the presence of more complete carcass portions in the EPPNB pit disproportionately affects the relative NISP of wild cattle in relation to the more even spatial distribution of other ungulate specimens, including goats [111]. This pit deposit shares similarities with another pit of wild cattle remains, Locus 1005 (also under the L1604 complex), previously described at KHH [11] and highlights the importance of *Bos* in ritual practice, which undoubtedly influenced the selection of taxa for provisioning the site during the EPPNB.

The important symbolic role of wild animals such as cattle has been established based on the art, figurines and burial goods found across the PPN *koine* (i.e. Göbekli Tepe and Çatalhöyük) [112–114]. Wild animal hunting likely held particular symbolic importance, which became especially poignant as the division of labor and labor scheduling were reconfigured during the development of animal management and cultivation [108,110,115,116]. Wild animal hunting may have sent a more costly signal of group membership than that of controlled animals during many types of rituals at KHH, such as feasts featuring the communal hunting of multiple dangerous wild cattle individuals [11]. Feasts and other rituals practiced at varying scales during the PPNB [117] were important for creating a sense of place and for integrating communities by maintaining traditions of shared symbolic practices with food [15,118,119].

## Supporting Information

S1 Table NISP of taxonomic groups represented at KHH by time period. Specimens derive only from well-dated contexts.
(DOCX)

S2 Table MNE of fused and unfused *Gazella* elements used to calculate age stages based on bone fusion [65,66]. Total gazelle MNE by phase: EPPNB n = 67, MPPNB n = 116, LPPNB n = 216.
(DOCX)

S3 Table MNE of fused and unfused *Capra* elements used to calculate age stages based on bone fusion [73]. (DOCX)

S4 Table MNE of fused and unfused *Bos* elements used to calculate age stages based on bone fusion [74]. (DOCX)

S5 Table MNE of fused and unfused *Sus* elements used to calculate age stages based on bone fusion [75]. (DOCX)

S6 Table Measurements included in *Capra* LSI for (A) EPPNB, (B) MPPNB, and (C) LPPNB assemblages at KHH (mm). Unfused bones marked with *.
(DOCX)

S7 Table Measurements included in *Bos* LSI from KHH (mm). Unfused bones marked with *.
(DOCX)
