# Supplementary material for: Provisioning the Ritual Neolithic Site of Kfar HaHoresh, Israel at the Dawn of Animal Management
Source: PLoS One. 2016 Nov 30;11(11):e0166573. doi: 10.1371/journal.pone.0166573 (PMC5130218; doi:10.1371/journal.pone.0166573)
Supplement: S1 Table — Specimens derive only from well-dated contexts. (DOCX) [file pone.0166573.s001.docx]

| **Taxon** | **EPPNB** | **MPPNB** | **LPPNB** | **Total** |
| --- | --- | --- | --- | --- |
| **UNGULATES** |  |  |  |  |
| Aurochs (*Bos primigenius*) | 112 | 77 | 89 | 278 |
| Aurochs-sized | 98 | 111 | 152 | 361 |
| Deer (Cervidae) | 1 | 2 | 1 | 4 |
| Fallow deer (*Dama mesopotamica*) |  | 2 |  | 2 |
| Wild boar (*Sus scrofa*) | 24 | 50 | 157 | 231 |
| Goat (*Capra sp.*) | 40 | 166 | 219 | 425 |
| Goat-sized | 99 | 194 | 265 | 558 |
| Roe deer (*Capreolus capreolus*) | 1 | 1 | 2 | 4 |
| Gazelle (*Gazella gazella*) | 171 | 568 | 876 | 1615 |
| Gazelle-sized | 309 | 831 | 1237 | 2377 |
| **CARNIVORES** |  |  |  |  |
| Fox (*Vulpes vulpes*) | 89 | 162 | 207 | 458 |
| Wild cat (*Felis silvestris*) | 18 | 65 | 70 | 153 |
| Indeterminate Canid (Canidae) | 1 | 1 | 1 | 3 |
| Marmot (*Marmota marmota*) | 2 | 3 | 6 | 11 |
| Medium Carnivore-sized | 27 | 123 | 194 | 344 |
| Indeterminate Mustelid (Mustelidae) |  |  | 3 | 3 |
| Pine Martin (*Martes foina*) | 1 | 1 | 3 | 5 |
| Polecat (*Vormela peregrusna*) | 4 | 5 | 5 | 14 |
| Small carnivore-sized | 3 | 4 | 1 | 8 |
| **SMALL GAME** |  |  |  |  |
| Cape hare (*Lepus capensis*) | 147 | 225 | 184 | 556 |
| Mediterranean spur-thighed tortoise (*Testudo graeca*) | 172 | 245 | 399 | 816 |
| Turtle (*Mauremys* or *Emys sp*.) | 2 | 13 | 27 | 42 |
| Snake (Indeterminate sp.) | 15 | 18 | 4 | 37 |
| Agamid lizard (*Agama stellio*) | 1 |  |  | 1 |
| Lizard (Indeterminate sp.) | 7 | 2 |  | 9 |
| Hedgehog (*Erinaceus europaeus*) | 2 | 7 | 1 | 10 |
| Fish (Indeterminate sp.) | 4 | 3 | 23 | 30 |
| Crab |  | 1 | 7 | 8 |
| Caucasian Squirrel (*Sciurus anomalus*) |  |  | 1 | 1 |
| Tiny Rodent | 3 | 2 | 5 | 10 |
| Small Rodent | 2 | 2 | 1 | 5 |
| Medium Rodent | 3 |  |  | 3 |
| Large Rodent | 1 |  |  | 1 |
| Murid rodent (Muridae) |  | 3 |  | 3 |
| Naked mole rat (*Spalax ehrenberghi*) | 9 |  | 2 | 11 |
| Vole (Microtinae) | 3 | 1 |  | 4 |
| Tiny Bird |  |  | 1 | 1 |
| Small Bird |  | 2 |  | 2 |
| Medium Bird | 12 | 10 | 13 | 35 |
| Partridge (*Alectoris chukar*) | 2 | 1 | 2 | 5 |
| Duck (*Anas platyrhynchos*) |  | 1 |  | 1 |
| Common coot (*Fulica atra*) |  |  | 2 | 2 |
| Rock dove (*Columba livia*) |  | 3 |  | 3 |
| Shark (Selachimorpha) |  | 1 |  | 1 |
| Shell |  | 1 | 3 | 4 |
| **BIRDS OF PREY** |  |  |  |  |
| Buzzard (*Buteo buteo*) |  | 4 | 4 | 8 |
| Indeterminate Falconiforme (Falconiforme) | 2 | 3 | 6 | 11 |
| Golden eagle (*Aquila chysaetos*) | 1 |  |  | 1 |
| Eurasian eagle owl (*Bubu bubo*) |  |  | 1 | 1 |
| Indeterminate owl (Strigidae) |  | 2 |  | 2 |
| Sparrowhawk (*Accipter nisus*) |  |  | 3 | 3 |
| Large Bird (*Buteo* or *Aquila* sized) | 7 | 1 | 5 | 13 |
| Huge Bird (*Gyps sp.* sized) |  | 3 | 1 | 4 |
| **Grand Total** | **1395** | **2920** | **4183** | **8498** |
