# Supplementary material for: Provisioning the Ritual Neolithic Site of Kfar HaHoresh, Israel at the Dawn of Animal Management
Source: PLoS One. 2016 Nov 30;11(11):e0166573. doi: 10.1371/journal.pone.0166573 (PMC5130218; doi:10.1371/journal.pone.0166573)
Supplement: S2 Table — Total gazelle MNE by phase: EPPNB n = 67, MPPNB n = 116, LPPNB n = 216. (DOCX) [file pone.0166573.s002.docx]

| Age Stage | Elements that Fuse at Stage | EPPNB | EPPNB | MPPNB | MPPNB | LPPNB | LPPNB |
| --- | --- | --- | --- | --- | --- | --- | --- |
|  |  | Unfused | Fused | Unfused | Fused | Unfused | Fused |
| Stage 1  *0-2 months* | Radius-Proximal | 0 | 2 | 0 | 3 | 1 | 9 |
| Stage 2  *3-7 months* | 1st Phalanx-Prox, 2nd Phalanx-Prox, Humerus-Distal, Pelvis-Acetabulum, Scapula-Glenoid | 5 | 24 | 6 | 53 | 11 | 87 |
| Stage 3  *8-10 months* | Tibia-Distal | 3 | 2 | 3 | 2 | 1 | 5 |
| Stage 4  *10-16 months* | Femur-Proximal, Calcaneum, Metapodial-Distal, Femur-Distal, Ulna-Proximal | 7 | 15 | 14 | 23 | 17 | 53 |
| Stage 5  *12-18 months* | Humerus-Proximal, Radius-Distal, Tibia-Proximal, Ulna-Distal | 4 | 5 | 4 | 8 | 12 | 20 |
