# Supplementary material for: Provisioning the Ritual Neolithic Site of Kfar HaHoresh, Israel at the Dawn of Animal Management
Source: PLoS One. 2016 Nov 30;11(11):e0166573. doi: 10.1371/journal.pone.0166573 (PMC5130218; doi:10.1371/journal.pone.0166573)
Supplement: S3 Table — (DOCX) [file pone.0166573.s003.docx]

| Age Stage | Elements that Fuse at Stage | EPPNB | EPPNB | MPPNB | MPPNB | LPPNB | LPPNB |
| --- | --- | --- | --- | --- | --- | --- | --- |
|  |  | Unfused | Fused | Unfused | Fused | Unfused | Fused |
| **I**  *0-6 months* | Radius-Proximal |  |  |  |  | 2 | 4 |
| **II**  *6-12 months* | Innominate-Acetabulum, Humerus-Distal, Scapula-Glenoid | 1 | 2 | 3 | 8 | 5 | 5 |
| **III**  *12-18 months* | 1st Phalanx-Prox, 2nd Phalanx-Prox | 1 | 3 | 2 | 6 | 2 | 10 |
| **IV**  *18-30 months* | Tibia-Distal, Metapodial-Distal | 1 | 2 | 1 | 3 | 10 | 6 |
| **V**  *30-48 months* | Radius-Distal, Femur-Proximal, Femur-Distal, Ulna-Prox, Tibia-Proximal, Calcaneum |  |  | 2 | 6 | 13 | 6 |
| **VI**  *>48 months* | Humerus-Proximal |  |  |  |  |  |  |
