# Supplementary material for: Provisioning the Ritual Neolithic Site of Kfar HaHoresh, Israel at the Dawn of Animal Management
Source: PLoS One. 2016 Nov 30;11(11):e0166573. doi: 10.1371/journal.pone.0166573 (PMC5130218; doi:10.1371/journal.pone.0166573)
Supplement: S4 Table — (DOCX) [file pone.0166573.s004.docx]

| Age Stage | Elements that Fuse at Stage | EPPNB  Unfused | EPPNB  Fused | MPPNB  Unfused | MPPNB  Fused | LPPNB  Unfused | LPPNB  Fused |
| --- | --- | --- | --- | --- | --- | --- | --- |
| **I**  *7-18 months* | Humerus-Distal, Radius- Prox, 1st Phalanx-Prox, 2nd Phalanx Prox | 1 | 4 | 1 | 3 | 7 | 9 |
| **II**  *24-36 months* | Tibia-Distal, Metapodial-Distal |  |  |  |  | 2 | 3 |
| **III**  *36-42 months* | Calcaneum |  |  |  |  |  |  |
| **IV**  *42-48 months* | Humerus-Proximal, Radius-Distal, Ulna-Prox, Femur-Proximal, Femur-Distal, Tibia-Proximal | 9 | 2 | 1 | 2 | 5 | 8 |
