# Supplementary material for: Provisioning the Ritual Neolithic Site of Kfar HaHoresh, Israel at the Dawn of Animal Management
Source: PLoS One. 2016 Nov 30;11(11):e0166573. doi: 10.1371/journal.pone.0166573 (PMC5130218; doi:10.1371/journal.pone.0166573)
Supplement: S5 Table — (DOCX) [file pone.0166573.s005.docx]

| Age Stage | Elements | Age for curve (months) | LPPNB  Unfused | LPPNB  Fused |
| --- | --- | --- | --- | --- |
| **I**  *0-8 months* | Scapula-Glenoid, Pelvis-Acetabulum, Radius-Proximal | 4 | 1 | 2 |
| **II**  *8-18 months* | Humerus-Distal, 2nd Phalanx-Proximal | 13 | 4 | 2 |
| **III**  *18-24 months* | Tibia-Distal, 1st Phalanx-Proximal, | 21 | 2 | 1 |
| **IV**  *24-36 months* | Metapodial Distal, Fibula-Distal | 30 | 2 | 1 |
| **V**  *36-48 months* | Calcaneum, Tuber calcis, Femur-Proximal | 42 | 0 | 0 |
| **VI**  *48-60 months* | Radius-Distal, Ulna-Proximal & Distal, Femur-Distal, Tibia-Proximal, Fibula-Proximal, Humerus-Proximal | 54 | 8 | 2 |
