# Supplementary material for: Provisioning the Ritual Neolithic Site of Kfar HaHoresh, Israel at the Dawn of Animal Management
Source: PLoS One. 2016 Nov 30;11(11):e0166573. doi: 10.1371/journal.pone.0166573 (PMC5130218; doi:10.1371/journal.pone.0166573)
Supplement: S6 Table — Unfused bones marked with *. (DOCX) [file pone.0166573.s006.docx]

A

| Element | Measurement [1] | EPPNB KHH *Capra* specimen measurements |
| --- | --- | --- |
| Calcaneus | GB | 20.11 |
| P1 | Bp | 12.97, 16.47 |
| Scapula | LG | 25.16 |
| Tibia | Bd | 33.71 |
| Ulna | BPC | 22.34 |

B

| Element | Measurement [1] | MPPNB KHH *Capra* specimen measurements |
| --- | --- | --- |
| Astragalus | BC | 23.42 |
| Femur | DC | 21.3* |
| P1 | Bp | 13.76, 15.35, |
| P1 | Bd | 12.57, 12.54, 12.72 |
| Radius | Bd | 33.11* |
| Radius | BFp | 29.66 |
| Tibia | Bd | 24.68* |
| Ulna | BPC | 26.46 |

C

| Element | Measurement [1] | LPPNB KHH *Capra* specimen measurements |
| --- | --- | --- |
| Astragalus | BC | 18.79, 19.17, 19.39, 19.39, 21.26 |
| Calcaneus | GB | 19.8, 22.2*, 25.77 |
| Femur | Bd | 39.87* |
| Femur | DC | 24.72, 25.61* |
| Humerus | Bd | 31.57 |
| Metacarpal | BP | 24.81 |
| Metatarsal | Bd | 24.72* |
| Metatarsal | BP | 20.1, 21.5, |
| Metatarsal | Bd | 28.81 |
| Phalanx 1 | Bd | 11.61, 11.93, 12.81, 12.97, 13.02*, 17.01, 14.48, 13.13*, 13.4*, 14.12 |
| Phalanx 1 | Bp | 12.51 |
| Radius | BFp | 28.51, 30.6*, 32.74*, 36.44 |
| Radius | Bd | 28.95*, 28.96*, 29.01, 30.36, 33.47* |
| Tibia | Bd | 26.11, 34.99 |
| Ulna | BPC | 21.79, 22.33*, 29.08 |
| Tibia | Bd | 33.94 |

1. von den Driesch A.  A Guide to the Measurement of Animal Bones from Archaeological Sites. Cambridge, MA: Harvard University Press; 1976.
