# Supplementary material for: Provisioning the Ritual Neolithic Site of Kfar HaHoresh, Israel at the Dawn of Animal Management
Source: PLoS One. 2016 Nov 30;11(11):e0166573. doi: 10.1371/journal.pone.0166573 (PMC5130218; doi:10.1371/journal.pone.0166573)
Supplement: S7 Table — Unfused bones marked with *. (DOCX) [file pone.0166573.s007.docx]

| Element | Measurement [1] | KHH specimen measurements |
| --- | --- | --- |
| Astragalus | GL | 84.39, 84.39 |
| Humerus | Bd | 91.11 |
| Metacarpal | Bp | 78.79 |
| Metatarsal | Bd | 73.4 |
| Nav. cuboid | GB | 64.27, 70.55 |
| Phalanx 1 | Bp | 27.53*,30.47*, 38.3 |
| Phalanx 2 | Bp | 30.34, 33.48, 34.1, 35.57, 36.25, 38.1, 38.54 |
| Radius | Bp | 85.35 |

1. von den Driesch A.  A Guide to the Measurement of Animal Bones from Archaeological Sites. Cambridge, MA: Harvard University Press; 1976.
